# Small Genomes and Big Data: Adaptation of Plastid Genomics to the High-Throughput Era

**DOI:** 10.3390/biom9080299

**Published:** 2019-07-24

**Authors:** Christen M. Klinger, Elisabeth Richardson

**Affiliations:** 1 Division of Infectious Diseases, Department of Medicine, University of Alberta, Edmonton, AB T6G 2R3, Canada; 2 Department of Biological Sciences, University of Alberta, Edmonton, AB T6G 2R3, Canada

**Keywords:** plastid biology, bioinformatics, biotechnology, next-generation sequencing

## Abstract

Plastid genome sequences are becoming more readily available with the increase in high-throughput sequencing, and whole-organelle genetic data is available for algae and plants from across the diversity of photosynthetic eukaryotes. This has provided incredible opportunities for studying species which may not be amenable to in vivo study or genetic manipulation or may not yet have been cultured. Research into plastid genomes has pushed the limits of what can be deduced from genomic information, and in particular genomic information obtained from public databases. In this Review, we discuss how research into plastid genomes has benefitted enormously from the explosion of publicly available genome sequence. We describe two case studies in how using publicly available gene data has supported previously held hypotheses about plastid traits from lineage-restricted experiments across algal and plant diversity. We propose how this approach could be used across disciplines for inferring functional and biological characteristics from genomic approaches, including integration of new computational and bioinformatic approaches such as machine learning. We argue that the techniques developed to gain the maximum possible insight from plastid genomes can be applied across the eukaryotic tree of life.

## 1. Introduction

### 1.1. Plastid Genomes

The endosymbiotic theory, proposed by Lynn Margulis in 1966, has provided the theoretical underpinnings of over five decades of endosymbiotic organelle biology research [1]. Through the interpretations of mitochondria and plastids as the enslaved remnants of free-living bacteria, scientists gained critical insight into the membrane biology, protein complement and genetics of these enigmatic organelles. One of the key traits supporting the identification of endosymbioses was the presence of organellar genomes, similar in structure, replication and expression to their prokaryotic cousins [2]. Critical insights into plastids and plastid-like organelles have arisen from close study of the plastid genome, aided by advances in sequencing technology. For example, gene sequencing of the 23S ribosomal gene in the apicoplast of the eponymous Apicomplexa were crucial to its identification as a highly reduced plastid-like organelle [3].

**Figure 1:**
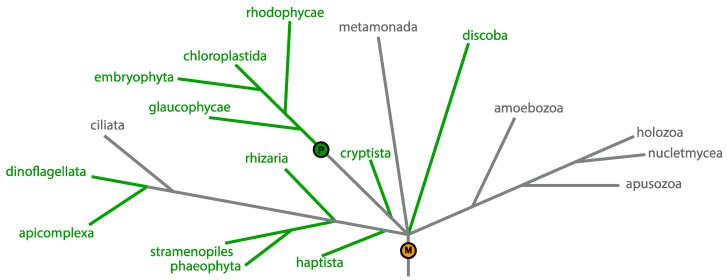
The diversity of eukaryotes, with lineages containing plastids identified in green. The origin of the cyanobacterial plastid present in the majority of photosynthetic organisms (excluding *Paulinella* spp., which has a plastid derived from a second primary endosymbiosis) and the origin of the alphaproteobacterial mitochondria are indicated by the green P and orange M, respectively.

Use of genes and genomics in biology has exploded in the past few decades. The phylogenetic species concept, where species were delimited based on % divergence in DNA sequence, introduced DNA as a unit of biological classification, and plant researchers increasingly turned to plastid genes to unravel the tangled web of algal and plant diversity [4]. The large subunit of the RuBisCO protein, essential for carbon fixation, is a commonly used marker gene for phylogenetic analysis of the relationships between plants and algae [5]. Plastids are relatively easy to extract from plant tissues, and contain an abundance of DNA; this, along with their uniparental maternal inheritance, makes them an ideal target for taxonomic gene sequencing [6]. To date, over 1000 plastid genomes have been sequenced from across eukaryotic diversity, including representatives from all known plastid-bearing phyla [7]. The array of genetic complements and structures revealed by this sequencing effort is astounding; from the tiny, highly-fragmented plastid genomes of dinoflagellates to the bloated, largely non-coding plastid genomes of Chlamydomonadales, plastid genomes can also have circular, linear, or multi-chromosomal structures [8], and the wide spread of plastids across eukaryotic diversity is shown in Figure 1. However, it is worth noting at this stage that plastids, generally, exhibit much less diversity in their genetic rearrangement than the other major genome-containing organelle, the mitochondria. The reasons for this are not known, though there is speculation that it is due to the near-ubiquity of mitochondria, all of which are derived from a single source long before the endosymbiosis of the first plastid, allowing greater genetic divergence to emerge over evolutionary time, also shown in Figure 1 [8].

### 1.2. Next-Generation Sequencing

The number of available plastid genomes has been massively increased by the advent of next-generation sequencing and the corresponding explosion in publicly accessible genetic information [7,9]. Experimental techniques using genetic sequencing, or a combination of methods including gene sequencing, have also become more and more prevalent [10]. Most journals have a requirement for genetic data produced during an experiment to be deposited in a curated sequence archive, and it is now common for candidates for new species to be identified not from microscopy or culture but from DNA sequence extracted from environmental samples. One of the most notable examples of this is the identification of the Asgardarchea, the closest extant archeal prokaryotic relatives of the eukaryotes [11,12]. Despite the extensive work that has been done to identify the genetic complement and architecture of Asgardarchea, none of them have yet been cultured or even photographed in detail [13]. It is also becoming more cost-effective to amplify an entire genome or transcriptome to identify a particular gene or pathway than to try to isolate the genetic components responsible for the phenotype [14]. With the flood of sequencing of DNA and RNA from environmental or RNA-seq experiments and subsequent publication of this data with minimal annotation, it is relatively easy to extract organelle genomes from these datasets [15]. Plastid genes can be extracted from RNA-seq datasets by searching for bacterial-like genes and plastid targeting sequences. It is therefore possible to assemble large, pan-taxon organelle genome datasets using just publicly accessible data [15]. These techniques have already been applied successfully to identify new plastid genomes from the Marine Microbial Eukaryote Transcriptome Sequence Project (MMETSP), which have provided insights for multiple photosynthetic lineages and their plastid gene complement [16].

**Figure 2:**
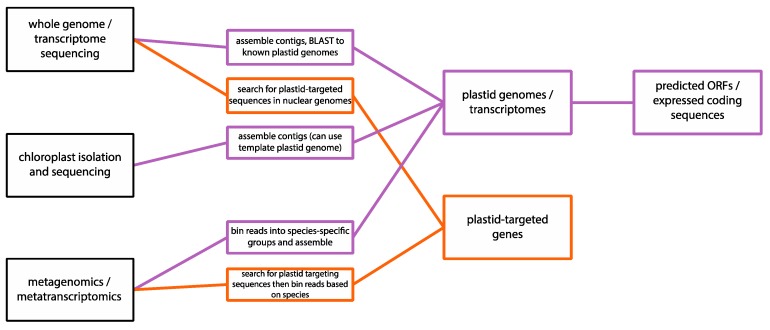
Methodology for identifying plastid genome sequences and plastid-targeted genes from various types of sequencing: genome/transcriptome sequencing projects, where the DNA is extracted from a cultured organism or single cell; chloroplast isolation and sequencing, where DNA is extracted from purified chloroplasts; and metagenomics/metatranscriptomics, where DNA from a microbial community is extracted and sequenced directly from an environmental sample.

The research potential of these datasets should not be underestimated; there are still many aspects of plastid biology that are known only in individual lineages, or the mechanisms for which have only been elucidated in selected model organisms. It is now possible for researchers, with extremely low up-front cost, to determine how widespread these traits are, and to use this information to inform their future research. Mechanisms that have been suggested to explain traits in specific species can now be evaluated across a much broader range of data and allow researchers to draw general conclusions on overarching plastid functional hypotheses across the entire diversity of eukaryotes—or, conversely, determine which functions are lineage-specific adaptations. A selection of common methods of obtaining DNA sequencing data relevant to plastids, and how the data can be analysed to extract both plastid genomes and plastid-targeting genes, is illustrated in Figure 2. There are dozens of genome and transcriptome assemblers available for research use, all of which use slightly different algorithms and assembly parameters and will be optimal for different experimental approaches. The development of genome assembly algorithms has been reviewed by Simpson and Pop [17], and more recently by Sohn and Nam [18]. Metagenomic and metatranscriptomic studies require an additional step before genome assembly where the combined environmental reads are separated by sequence; similarly, many programmes are publicly available and optimised for various types of data, reviewed in Ladoukakis et al. [19]. Finally, genes and open reading frames (ORFs) can be predicted from high-throughput sequencing data by codon analysis, as regions with long stretches of coding amino acids between a START and STOP codon can be further analysed for similarity to known genes by using algorithms such as BLAST [20]

Here, we present two case studies where researchers have evaluated lineage-specific plastid genetic traits over a large dataset assembled from publicly available data. This has allowed functional hypotheses based on specific plastid genomes to be evaluated over plastids generally within that lineage, or across photosynthetic diversity. We then use these examples to examine how bioinformatic tools created to analyse enormous genetic datasets have been invaluable for this effort. We also suggest other research techniques which could add to bioinformatic re-analysis of publicly available data in fields as diverse as evolutionary cell biology, ecology, and biotechnology.

## 2. Case Studies

### 2.1. Case Study 1: Codon Usage

Codon usage bias arises as a consequence of redundancy within the genetic code for almost all amino acids; those that can be encoded for by between two and six codons (two- and six-fold redundancy, respectively), are said to have synonymous codons. Under a random null model, all synonymous codons would be expected to be present in protein coding genes at equal frequency. Codon usage bias therefore refers to deviations from this null [21]. Implicit in the genetic code is the fact that third position substitutions are frequently synonymous, a fact which allows overall genome composition (GC-richness) to play a substantial role in shaping increased representation of NNA/NNT over NNC/NNG codons when the amino acid coded is synonymous [22,23]. This ‘mutational hypothesis’ is offset by the ‘translational hypothesis’ that posits that codon usage is optimized in highly expressed genes to improve either translation speed, translation efficiency, or both [21,24].

Support for the translational efficiency hypothesis comes from a multitude of observations. In bacteria, although overall patterns follow compositional biases, growth rate was found to correlate with codon usage of highly expressed genes [25]. Similarly, codon composition was found to differ in highly expressed genes in diverse plastids across multiple lineages [26,27]. However, the relevance of translational efficiency in endogenous genes has been questioned by the notion that transcription initiation, rather than elongation, is typically rate-limiting for protein synthesis [24]. With expression of transgenes, where the overexpression of heterologous mRNA often exceeds endogenous mRNAs by several-fold, codon optimization has been shown to increase expression by up to 1000-fold [28].

Regardless of the exact mechanisms governing codon bias, a recent study highlighted the extent to which the phenomenon exists across 103 publicly available plastomes by using a combination of previously described metrics and resampling simulations. Their results were variable, but despite the lack of overall strongly observed trends, codon bias was found to be highly lineage-dependent and to slightly favour highly expressed genes [23]. Other studies have shown that codon usage patterns can be a confounding factor in identifying trends if not considered properly [29]. Within the photosynthetic dinoflagellates, the ancestral plastid is of red algal origin and contains the pigment peridinin. However, this plastid has been serially replaced on multiple occasions, including through the putative engulfment of a haptophyte giving rise to a 19′ hexanoyloxyfucoxanthin-type plastid (commonly referred to simply as a fucoxanthin plastid). As such, it is expected that haptophyte and fucoxanthin plastid genes should place as sisters in phylogenetic analysis. However, DNA-based phylogenies of *psaA* and *psbA* appeared to suggest that haptophytes instead formed an outgroup to all dinoflagellate sequences, with the fucoxanthin plastid as ancestral, rather than secondarily acquired. Extensive re-analysis of datasets was conducted where Leu, Ser, and Arg codons, the only amino acids with codon variation at the first codon position, were either omitted or recoded. These results, alongside additional protein-based phylogenies, showed that the strong relationship between peridinin dinoflagellates and haptophytes was a result of codon bias. This suggests that the haptophyte + all dinoflagellate relationship in DNA phylogenies was artefactual and was due to shared codon composition bias between a subset of peridinin-containing dinoflagellates and haptophytes [29]. Hence, codon composition bias will be important to study moving forward, both as a means to better understand the evolutionary implications of plastid evolution, but also as a means for the expression of heterologous gene products in algae and beyond.

### 2.2. Case Study 2: RNA Editing

RNA editing is a common post-transcriptional modification frequently found in mitochondria of diverse eukaryotes, but occasionally in the nuclear and plastid genomes as well [30]. Some plastomes of land plants are heavily edited [31], but the majority of species show a comparatively lower editing rate, at or below 1% of positions within coding sequences. Organellar C-U interconversions are observed across land plants, but not in green algae. Although the complete machinery of the editing complexes and the nature of the editing mechanism are still being elucidated, it appears that editing sites are specified by pentatricopeptide repeat (PPR) proteins [32]. This specificity of PPR proteins has allowed the construction of databases and the development of predictive tools for PPR binding sites.

**Figure 3:**
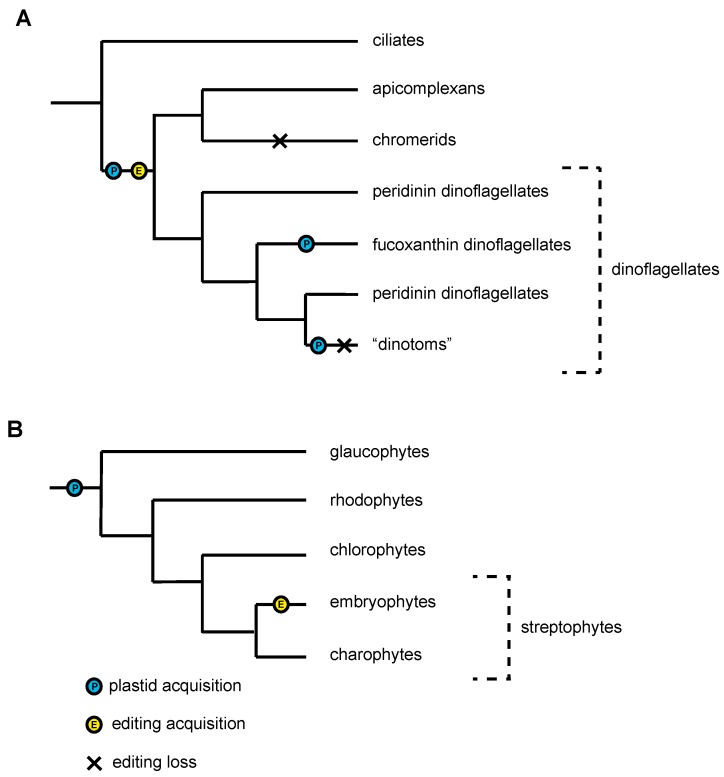
Convergent evolution of RNA editing in plastids. (**A**) RNA editing in the alveolates, including the Apicomplexa and dinoflagellates. There have been two additional plastid acquisitions in dinoflagellates, indicated by the blue Ps: the uptake of a haptophyte-derived plastid in the fucoxanthin dinoflagellates and the uptake of a diatom-derived plastid in the “dinotoms”. The emergence of transcript editing at the base of the Apicomplexa and dinoflagellates is indicated with a yellow E. Dinotoms do not appear to exhibit transcript editing, while the trait has been retained in fucoxanthin dinoflagellates. This trait also does not appear to be present in chromerids, the closest apicomplexan relative with a retained photosynthetic plastid. These losses of transcript editing are indicated with black crosses. (**B**) RNA editing in the Archaeplastida, with the primary endosymbiosis of a cyanobacteria at the base of the clade indicated with a blue P. Restricted (U to C and C to U) RNA editing in this clade appears to be restricted to the land plants, and the emergence of this trait is indicated by a yellow E.

Plastid RNA editing is also found in alveolates, in a case of what appears to be convergent evolution (shown in Figure 3A,B respectively). The alveolate group dinoflagellates possess often extensive plastid RNA editing, with overall rates up to ~5% [33]. Functional explanations for these editing events mostly evoke the removal of premature STOP codons or some effect on base and/or codon composition [34,35,36,37,38]. Studies focusing on individual lineages in isolation over a decade failed to find consistent patterns across dinoflagellates. In the case of *Symbiodinium minutum*, it was also suggested that changing the hydrophobicity of encoded proteins might be important. A systematic study, incorporating both novel and publicly available data and making use of computer simulations, found general trends such as clustering of editing events and propensity for editing to improve biochemical similarity of homologous amino acids to those from lineages that do not undergo editing, but failed to identify any putative signals directing editing events [33]. The mechanism of RNA editing in dinoflagellates remains to be uncovered, although the low-level nuclear RNA editing detected in *S. microadriaticum* was associated in some cases with significantly enriched motifs and genes encoding PPR proteins were detected in the genome [39]. This suggests an editing protein complement similar to that of land plants. RNA editing has also been identified in Apicomplexa, a sister group to dinoflagellates, which retain a remnant plastid, along with a single apicoplast-targeted PPR protein [40].

Mechanistically, RNA editing can be envisioned either as a directed process, in which an encoded factor (for example, protein and/or RNA) directs a specific nucleotide alteration at a specific position (call this a “editing-directing factor”, EDF) based on the genomic context, which could be the adjacent sequence, higher-level structure, or some other defining feature. Alternatively, editing could be promiscuous, either by reducing the stringency with which cellular machinery recognizes editing sites or by having a large number of EDFs that are capable of duplicating, accumulating mutations and novel features, and, hence, altering their specificity.

RNA editing presents a challenge to adaptive evolutionary theory, given the presence of silent editing events (i.e., those that do not change the encoded amino acid), editing events that move away from biochemical consensus, and editing events in non-coding regions, which may or may not have any functional implications [29,30,31,32,33,37,38,39,40,41]. In land plants, it has been suggested that changes in the subunit composition, and hence subunit–subunit interactions, of the plastid NDH complex may have been eased by RNA editing, while in *S. microadriaticum* it appears that nuclear RNA editing may be responsive to stress conditions [39,42]. In dinoflagellates in general, plastid RNA editing has been suggested as a mechanism for correcting deleterious mutations induced by the high level of sequence evolution in the plastid genome [43].

**Figure 4:**
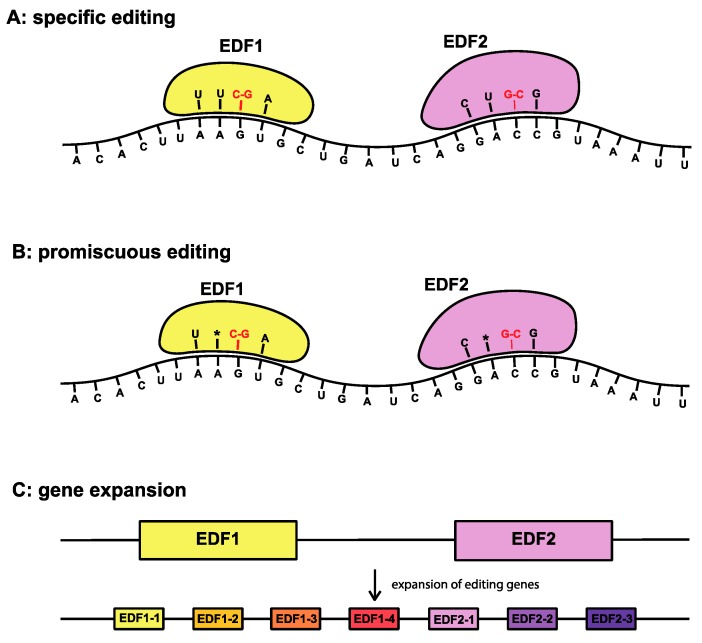
Possible models of transcript editing with editing factors (EDFs). (**A**) Specific editing, where specific EDFs are responsible for facilitating edits on matching regions of the transcript. (**B**) Promiscuous editing, where there is some flexibility in the possible matches that any one EDF can make, or what conversions are possible. (**C**) Gene expansion, where a small group of ancestral EDFs expand within the genome and evolve different specificities to transcript sequences or possible base conversions.

In general though, the origin of such adaptive hypotheses for RNA editing is difficult to conceive, at least in cases where a nucleotide change that could be subject to editing induces a lethal alteration, as this would require both general editing machinery and an EDF capable of reverting this change be present and expressed prior to the change actually occurring for the organism to survive. As discussed above, this could occur either through the relative promiscuity of each individual EDF, through the presence of a large complement of EDFs in an organism’s genome, or a combination thereof. Three potential editing scenarios are indicated in Figure 4. Figure 4A shows a method of RNA editing which would result in specific edits with a relatively small number of base pair conversions, which corresponds to the editing observed in land plants. Figure 4B,C indicate two possible scenarios for more promiscuous editing, both in terms of editing site selection and potential base pair conversions. Promiscuous editing could be problematic, as the advent of an EDF may itself induce an undesirable change (i.e., from a nucleotide to one that causes a nonsynonymous change or encodes a premature STOP codon), but it is likely that such a change would not be driven to fixation and hence, on the whole, the system would retain only generally positive events. This is consistent with the pattern of editing found in dinoflagellates. In *Plasmodium*, editing appears to be stage-specific, which implies additional developmental functions [40].

Regardless of the exact nature of the editing landscape in plastids, it is likely an irremediable genetic feature. Over time, the ability of editing to correct multiple deleterious nucleotides at the genomic level would lead to an increasingly low probability that all such bases could revert to the ancestral, or otherwise synonymous, base. Failing this, editing becomes an essential function for the organism, despite arguably greater complexity and the energetic requirements to maintain and express editing machinery. Additionally, the presence of editing machinery, especially as a promiscuous system, would allow for additional deleterious changes at the genomic level over time, especially if the evolutionary rate were high, as has been described in dinoflagellates [33]. This non-adaptive or neutral theory is consistent with the concept of an “evolutionary ratchet”, which suggests that the dependence of a cellular system that has accumulated mutations (here, the expression of genes) on another system (here, RNA editing) means that reversal to independence is unlikely [44]. Continued study of RNA editing using larger datasets and more sophisticated analytical and statistical tools will likely improve our understanding of this fascinating molecular feature.

## 3. Future Possibilities

### 3.1. Evolutionary Biology

Some of the most interesting debates in evolutionary cell biology revolve around the evolutionary origins of plastids. The history of plastid transfer, gain, and loss is an unbelievably complex web of serial endosymbiosis that can often seem hopelessly tangled, particularly within the group historically identified as the “chromalveolates”, or organisms containing red algal-derived plastids [45,46]. The exact relationships between these organisms and their chosen endosymbionts are an area of lively debate—one which we have no desire to re-open—but has resulted in extremely active interest in and development of sophisticated tools to detect the evolutionary origins of specific plastid genes [47]. Endosymbiosis, and the associate gene transfer, is a unique way of introducing genetic plasticity and many hypotheses for the evolution of extant photosynthetic diversity which incorporate endosymbiosis as an evolutionary mechanism have not yet been tested across the diversity of eukaryotes, or deeply within that diversity. For example, the “limited transfer window” hypothesis for horizontal gene transfer between organelles within an organism predicts that the fewer plastids an organism has, the less likely transfer is to the nucleus or other organelles. This hypothesis was proposed in 2006 by Barbrook et al. [48] and was tested extensively across plastid diversity in 2011 by Smith et al. [49,50]. Since 2011, nuclear and plastid genome pairs have been acquired from a multitude of organisms, including those adapted to extreme environments such as the High Arctic and from additional eukaryotic groups such as the excavates and chlorarachniophytes [51,52,53]. Research on the genomes of the chlorarachniophyte *Bigelowiella natans* and the excavate *Euglena gracilis* both reported gene transfer between the organelles consistent with the limited transfer window hypothesis [52,53]. Expanding the techniques used in Smith et al. [49,50] into this wider range of organisms across eukaryotic diversity and incorporating bioinformatic models of different selective pressures on this trait could provide additional insight into this long-held hypothesis.

### 3.2. Ecological Research

Plastids are an essential aspect of photosynthesis, and therefore an essential part of global nutrient cycling [54]. Some of the clades identified as most important for carbon fixation in the open ocean, such as the dinoflagellates algae and diatoms, have rich evolutionary histories for both their nuclear and their plastid genomes [43,55]. Modern molecular ecology questions have increasingly focused on the functional consequences of identifying a particular taxon or genetic pathway in an environment. For example, if many parasitic lineages are identified in a metatranscriptomic study of soils, what does this say about the nutrient cycling within an environment [56]? If we are able to determine the microbial community present in an environment and have previously determined which functional traits are associated with the plastids of these organisms, then researchers can begin to make conclusions about the ecological consequences of those traits being active. We also briefly discussed in the introduction the potential identification of organelle sequences and genes from environmental DNA sequence runs; in most cases, organelle-derived DNA is discarded from eukaryotic microbial community assessments as the 18S rRNA gene is used as a proxy for diversity [57]. However, this genetic data may contain additional information that can be incorporated into diversity assessments. For example, the most commonly used primers for amplifying eukaryotic DNA from environmental DNA, the universal eukaryotic primers for the V4 region of the 18S rRNA developed in Stoeck et al. [58], have been demonstrated to amplify excavate DNA relatively poorly [59]. However, some excavates, including the group Euglenozoa, contain functional plastids [7]. Incorporating organelle DNA into environmental surveys may assist in identifying excavates whose presence may otherwise be overlooked. This has already been used successfully in a survey of the fungal and plant communities of a protected wetland in Wood Buffalo National Park, Alberta, Canada. Porter et al. [60] extracted nuclear ITS spacer region sequences and plastid *rbcL* sequences from metagenomic samples and used them to determine the diversity of local fungi and plants, respectively.

Plastid genomics, particularly from environmental DNA studies, can also be used to identify if a plastid genome itself is adapted to an environment. Genome-level evolutionary adaptation to environments has long been observed. For example, patterns of gene loss and genome reduction in parasitic lineages have long been observed across the diversity of eukaryotes [61]. One could expect to identify adaptations that may provide a selective advantage in the organelles of organisms extracted from extreme environments. We attempted, albeit unsuccessfully, to use this approach when examining the genomes of obligate psychrophile algae isolated from the High Arctic. Using the reasoning that the genomes of these organisms would be exposed to higher than usual amounts of UV radiation, we hypothesised that their plastid genomes would use fewer codons containing thymine repeats, which could result in the production of thymine dimers. An automated methodology for detecting thymine dimers was initially proposed to detect internal poly(T) gene sequences in Klinger et al. [33]. Due to technical difficulties, we were unable to identify any definitive trends, but this illustrates the lines of research that can be tested through bioinformatic analysis of plastid genome data. Mining public data archives for plastid genomes may also result in the identification of previously unheard-of plastid traits; the discovery of the cercozoan *Paulinella* spp. and the sequencing of its plastid genome revealed that a secondary primary endosymbiosis had occurred in this lineage, where previous hypotheses suggested a single primary endosymbiotic origin of both the plastid, in the same way the mitochondria is accounted for by a single primary endosymbiosis [62].

### 3.3. Incorporation of New Computational Techniques and Biotechnology

With the amazing possibilities generated by bioinformatic analysis of plastid genomes across eukaryotic diversity in front of us, it is worth considering how this approach could develop in the light of rapidly advancing technology [10]. Machine learning and artificial intelligence are increasingly being incorporated into genomic analyses, with machine learning algorithms and tools being developed which are able to, for example, identify ORFs, enhancer and suppressor elements, and protein binding sites [63,64]. In these techniques, machine learning algorithms compare large datasets of different states: for example, gene/not gene, diseased/not diseased, or regulatory element/not regulatory element. The algorithm can identify features that are common to only one type of dataset or the other, and then use these to classify new examples. The major benefit of these techniques is that they are affected to a much lesser extent by the biases inherent in the identification of these sites by manually developed tools; machine learning algorithms are able to identify one condition versus the other often with more sophistication than trained experts [65]. One example from Case Study 2: RNA editing shows an obvious potential application of this technology: there are already algorithms based on machine learning that are able to identify putative editing sites in plant plastids [66,67]. If given enough examples of edited and non-edited sites in dinoflagellates, where the editing machinery likely evolved independently, would these algorithms be able to correctly classify edited versus non-edited transcripts? Machine learning could also be incorporated in examination of the “limited transfer window” hypothesis discussed in the previous paragraph: The studies in Smith et al. [49,50] provided a wealth of examples of horizontally-derived nuclear and mitochondrial sequences which could be used to train a classifier to identify transfers automatically across a much larger dataset, or identify more cryptic transfers. This is an exciting line of enquiry, particularly in organisms where molecular tools for genetic manipulation are absent or newly developed, such as dinoflagellates.

Synthetic biology is another area where plastid genomics using pan-eukaryotic datasets may be extremely effective. Processes associated with plastids, and in particular carbon fixation, have become even more critical for us to understand in the context of ongoing climate change [68]. There is already considerable effort being placed into developing synthetic organisms capable of mitigating some forms of anthropogenic change, such as degradation of oil spills [69]. By identifying trends in codon usage and distribution of metabolic pathways across plastids, researchers can identify which genes, codons, or genetic motifs are essential—or even just most efficient—in a synthetic organism. Bioremediation and biofuel production, both of which involve manipulation of hydrocarbon chains by organisms to produce a useful output or degrade an undesirable one, already make great use of photosynthetic organisms and plastid pathways [70]. For example, plastid genome editing in the marine algae *Nannochloropsis oceanica* can increase fatty acid biosynthesis and therefore biofuel production [71].

## 4. Conclusions

In the early twenty-first century, all scientific disciplines are in some respect governed by the incredible acceleration of technological development. In this review, we discuss how the co-advancement of two aspects of computational biology, bioinformatics and next-generation sequencing, have opened up an entirely new approach for studying plastid genomics and cell biology. We illustrate two cases in which mining publicly available databases to isolate plastid genome sequences has allowed researchers to implement bioinformatic approaches to test functional hypotheses across large-scale, pan-lineage datasets. We have also provided some examples of how this approach may open doors for further research in several disciplines, including ecology, synthetic biology and evolutionary biology. We eagerly await further developments in computational biology that will allow even deeper understanding of the genetic possibilities present in possibly the most intriguing cell structure: the plastid.
